# A prospective, randomised, controlled, double-blind phase I-II clinical trial on the safety of A-Part^® ^Gel as adhesion prophylaxis after major abdominal surgery versus non-treated group

**DOI:** 10.1186/1471-2482-10-20

**Published:** 2010-07-06

**Authors:** Reinhold Lang, Petra Baumann, Karl-Walter Jauch, Claudia Schmoor, Christine Weis, Erich Odermatt, Hanns-Peter Knaebel

**Affiliations:** 1 Department of Surgery, University Munich-Großhadern, Marchioninistrasse 15, 81377 Munich, Germany; 2 Aesculap AG, Department of Clinical Science, Am Aesculap Platz, 78532 Tuttlingen, Germany; 3 Clinical Trials Centre, Elsässer Strasse 2, 79110 Freiburg, Germany; 4 Aesculap AG, R&D Closure Technologies, 78532 Tuttlingen, Germany

## Abstract

**Background:**

Postoperative adhesions occur when fibrous strands of internal scar tissue bind anatomical structures to one another. The most common cause of intra-abdominal adhesions is previous intra-abdominal surgical intervention. Up to 74% of intestinal obstructions are caused by post surgical adhesions. Although a variety of methods and agents have been investigated to prevent post surgical adhesions, the problem of peritoneal adhesions remains largely unsolved. Materials serving as an adhesion barrier are much needed.

**Methods/Design:**

This is a prospective, randomised, controlled, patient blinded and observer blinded, single centre phase I-II trial, which evaluates the safety of A-Part^® ^Gel as an adhesion prophylaxis after major abdominal wall surgery, in comparison to an untreated control group. 60 patients undergoing an elective median laparotomy without prior abdominal surgery are randomly allocated into two groups of a 1:1- ratio. Safety parameter and primary endpoint of the study is the occurrence of wound healing impairment or peritonitis within 28 (+10) days after surgery. The frequency of anastomotic leakage within 28 days after operation, occurrence of adverse and serious adverse events during hospital stay up to 3 months and the rate of adhesions along the scar within 3 months are defined as secondary endpoints. After hospital discharge the investigator will examine the enrolled patients at 28 (+10) days and 3 months (±14 days) after surgery.

**Discussion:**

This trial aims to assess, whether the intra-peritoneal application of A-Part^® ^Gel is safe and efficacious in the prevention of post-surgical adhesions after median laparotomy, in comparison to untreated controls.

**Trial registration:**

NCT00646412

## Background

Post surgical adhesions are quite common, i.e. they can affect up to 93% of patients undergoing abdominal surgery [1,2]. Adhesions are internal scars developing after trauma and involving the injured tissue and the peritoneum [3]. The magnitude of the problems caused by adhesions was highlighted by several studies [4-7]. Depending on the location and the structure of the adhesion serious complications may be caused thereby, such as large and small bowel obstruction. Pelvic adhesions are associated with infertility in 15-20% of cases [8]. A number of products that help to reduce or to prevent tissue adhesion are marketed [2]. Barrier materials in various forms such as films, viscous gels and intra-peritoneal solutions have been used clinically for the prevention of surgical adhesions. However, these materials have had limited success, and no treatment is adopted so far as a standard therapy [9].

### Rationale

Several materials and methods have been tested for the prevention of post surgical adhesions, but the problem is still unsolved [10-12]. The most established method in adhesion prevention is to apply a barrier between the wounded surfaces. Barrier materials should be easy to apply and should remain in place for several days to allow serosal re-epithalization and should be resorbed afterwards and excreted without systemic reactions or inappropriate accumulation. Materials should be non-inflammatory and non-reactive and should not interfere with the healing processes of sutures, anastomoses or the incision.

### Purpose

A-Part^® ^Gel [13] a bioresorbable transparent gel composed of polyvinylalcohol (PVA) and carboxymethylcellulose (CMC) was developed to meet these features mentioned above. Both components PVA and CMC are already used for various biomedical applications [14-17]. The established use of PVA and CMC has confirmed their good biocompatibility [10,18-25]. Material characteristics and preclinical data of animal studies have established the promising properties of A-Part^® ^Gel as a new measure against post surgical adhesions [26,27]. Therefore this prospective, randomised, controlled, patient and observer blinded trial was designed as a first evaluation of the safety of A-Part^® ^Gel as an adhesion prophylaxis after abdominal surgery in comparison to a non treated control group in man.

## Methods/Design

### Objectives of the study

The aim of this study is a first assessment of the safety of A-Part^® ^Gel applied as an adhesion prophylaxis after major abdominal surgery, by specific observation of two major complications of abdominal surgery; wound healing impairment or peritonitis; in comparison to a non treated control group (primary objective) within 28 (+10) days after surgery (for definition see table 1). The incidence of adverse events and serious adverse events occurring 3 months postoperatively, with special attention to anastomotic leakage, between the two treatment groups, will be used to further consider the safety of A-Part^® ^Gel. To explore the efficacy of A-Part^® ^Gel in reducing post-surgical adhesions, ultrasound examination will be performed up to 3 months postoperatively (secondary objectives).

**Table 1:** Diagnostic criteria of complications

Complication	Diagnostic criteria/Definition
**Impaired wound healing**	
a)Delayed wound healing	• Necrosis of wound edge
	• Dehiscence
	
b) Development of surgical site infection	• Purulent secretion from the wound
	• Germ organism isolated from an aseptically obtained culture of fluid or tissue from superficial incision
	• Local signs of infection or systemic signs (fever, leukocytosis, rising CRP) without other plausible causes (e.g. pneumonia, urinary tract infection)
	• Diagnosis of an abscess in deep soft tissue (an abscess in deep soft tissue is defined as microbiological verification of suspect specimen which is generally taken by means of ultrasonic puncture)
	
**Peritonitis**	• fever, leukocytosis, abdominal pain, muscular defense, absence of bowel sounds, metabolic disturbances or severe hypotension with multiple organ failure (MOF)
	Diagnosis confirmed by:
	▪ Fluid collection by puncture or drainage
	▪ Sonography
	▪ Computed tomography
	▪ Re-Intervention/Revision
	
**Anastomosis leakage**	• Fever, abdominal pain, metabolic disturbances, intra-abdominal abscesses, or sepsis with multiple organ failure (MOF)
	Diagnosis confirmed by:
	▪ Fluid collection by puncture or drainage
	▪ Sonography
	▪ Computed tomography
	▪ Colonic contrast enema (water soluble contrast medium)
	▪ Re-Intervention/Revision

The incidence of adhesions between the visceral organs and the abdominal wall will be diagnosed by ultrasound examination using a linear-array transducer. The scar of the abdominal incision will be divided into equal parts of 2,5 cm, starting 2 cm from the cranial beginning of the scar. At each assessment point spontaneous visceral slide (produced by regular and deep respiration motion) will be examined using ultrasound scanning. Reduced movement following respiration will count as restricted visceral slide, i.e. adhesions. The total number of assessment points and the number of assessment points with adhesions will be documented.

#### Primary endpoint

- Occurrence of wound healing impairment or postoperative peritonitis within 28 (+10) days after surgery.

Impaired wound healing is defined as delayed wound healing or the development of surgical site infection.

Delayed wound healing has to be diagnosed if at least one of the following criteria is fulfilled:

- Necroses of wound edges occur.

- Primary or secondary dehiscence occurs (primary dehiscence is defined as retreating of wound edges immediately after surgery; secondary dehiscence is defined as retreating of wound edges after start of the wound healing.

Surgical site infection and postoperative peritonitis has be assumed as present, if one or more of the criteria which are shown in table 1 are fulfilled.

#### Secondary endpoints

- Occurrence of anastomosis leakage within 28 (+10) days after surgery.

- Occurrence of Adverse Events (AE) and Serious Adverse Events (SAE) during the postoperative hospital stay and up to 3 months after surgery.

- Adhesion rates along the scar after 14 days, 28 (+10) days and 3 months (±14 days) days examined by ultrasound.

For the definition of anastomosis leakage please see table 1. Assessment of adhesion development will be done by ultrasound assessment. Sigel *et al. *described a technique for non-invasive ultrasound examination to detect and map abdominal adhesions. The findings that identify abdominal wall adhesions are based on the presence or restriction of ultrasonically observed movement of abdominal viscera in reference to abdominal wall.

### Design

**Figure 1:**
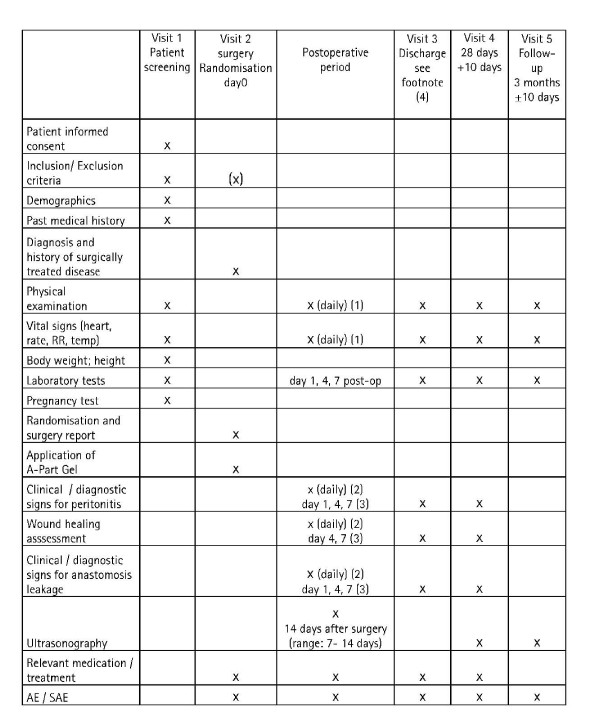
**Flow chart of treatment schedule**. (1) Daily documentation until day 7 has to be done in the patient chart. CRF documentation will be done on day 1, 4, ±1 and 7 + 1 after surgery. Adverse events have to be documented as soon as they occur. (2) Has to be done daily with respective documentation in the patient file. (3) CRF documentation will be done on these days. (4) Generally if the patient is discharged before day 7, all documentation required until the day of discharge (e.g. day 1, 4, etc.) has to be completed together with the discharge page (visit 3). However if discharge is on day 1, 4 + 1 or 7 + 1 the documentation of the respective day (day1, 4 or 7) can be omitted (with the exception of the header which has to be filled in); only the discharge page (visit 3) needs to be filled in instead.

This is a prospective, randomised, controlled, double-blinded, single centre study. Patients will be blinded as well as the evaluating physician conducting the ultrasound examination. In case of a non sufficient recruitment this study can be expanded into a multicentre trial. Patients enrolled in the trial will be randomly allocated either to the treatment group receiving A-Part^® ^Gel or to the untreated control group (for inclusion and exclusion criteria see table 2). Randomisation will take place during the operation. Initially there will be a staggering of the treatment of the patients, so that during the early phase of the study there is a one-by one exposure of patients to A-Part^® ^Gel. Both groups consist of 30 patients. After discharge from the hospital the investigator will examine the patients at 28 days (+10) and after 3 months (±14 days) after surgery (figure 1).

**Table 2:** Eligibility Criteria

Inclusion criteria	Exclusion criteria
• Patients of both sexes undergoing a primary elective median abdominal incision	• Patients with a known history of adhesion or peritonitis
• Age equal or greater than 18 years	• Patients with a known sensitivity to polyvinylalcohol or carboxy-methylcellulose
• Written informed consent	• Simultaneous participation in another clinical trial with interfering end-points
• Expected incison length ≥15 cm	• Emergency surgery
• Expected survival time more than 12 months	• Patients with peritoneal carcinosis or peritoneal dialysis
• For female adults of reproductive potential: negative pregnancy test at visit 1 and sufficient contraception from time of written consent up to at least 4 months	• Patients with systemic immunsuppression (e.g. hydrocortisone >50 mg per daily, other immunsuppressants like Azathropin, Mycophenolatmofetil, Ciclosporin, Everolimus, Methotrexat ect.), chemotherapy or radiotherapy within the last 2 weeks prior surgery
	• Patients with ascites >200 ml
	• ASA > 3
	• Patients with intra-abdominal abscess or other intra-abdominal infection
	• Renal impairment (Creatinine >1.3 mg/ml)
	• Pregnant or breast-feeding women
	• Lack of compliance
	• Surgical procedures or patients which require insertion more than 2 intra abdominal drains

### Eligibility

Detailed inclusion and exclusion criteria are specified in table 2. Patients are screened consecutively for eligibility in the participating centre after approval of the study protocol by the local ethics committee of the Ludwig-Maximilians-University (LMU), Munich, Germany. A contract has been signed by Aesculap AG and the participating centre for correct conduction of the trial according to Good Clinical Practice. The participating surgeons performing the intervention have been instructed by detailed manuals.

### Consent

The participating centre recruits trial patients from its patients who are scheduled for an elective primary median abdominal incision with a expected length of ≥15 cm. All patients who seem to fit to the in- and exclusion criteria will be asked whether they are willing to participate in the trial and they will be informed about the purpose of the trial, the operation modalities, data management, and their possibilities and risks. Interested patients will be screened according to inclusion and exclusion criteria and included into the trial after written Informed Consent has been obtained from the patient.

### Randomization

Block randomisation with randomly varying block sizes and a ratio 1:1 to the two treatment arms will be used in order to provide treatment groups of approximately equal sizes. The block length will be documented separately and will not be disclosed to the investigators. Randomisation code was generated by the statistician of the Clinical Trials Centre (ZKS), Freiburg, Germany. To guarantee concealment of the randomisation, it will be performed using sealed opaque envelopes which were produced by the ZKS and are kept at the study centre. Randomisation will take place before abdominal wall closure. If there is an explicit need to unblind during the follow up, the randomisation envelopes can be used to unblind the patient.

### Intervention

After the surgical procedure, the abdominal incision is closed from the caudal and cranial end up to the middle with a monofilament suture loop by using the continuous suture technique. Up to 3 cm of the incision will be left open in the middle of the incision. A-Part^® ^Gel is prepared and brought under the incision through the open part in the middle. A-Part^® ^gel syringe is placed to one end of the incision. The A-Part^® ^Gel is applied along the partly sutured incision while pressing out slowly the viscous gel onto the abdominal wall. This procedure is repeated on the other half of the incision, and when the syringe is taken out from the open middle part of the incision. Thereafter the abdominal incision is completely closed according to the INSECT suture technique [28] and the skin closure is performed with staples. It is recommended to use 10 ml of A-Part^® ^Gel for covering 10 cm of incision. The A-Part^® ^Gel is only applied by a trained surgeon.

### Study Device

A-Part^® ^Gel is a sterile, absorbable, translucent adhesion barrier composed of two unmodified water soluble biocompatible polymers: polyvinyl alcohol (PVA) and carboxymethylcellulose (CMC) [13,26]. Both components PVA and CMC are already used for various biomedical applications [10,16,17,22-25]. PVA is used in drug coating, contact lenses, tendon repair or artificial articular cartilage [14,15]. CMC is used as an additive in tablets, suspensions and creams. PVA is biologically inert and is not degraded in mammal metabolism [29,30]. As a water soluble polymer, PVA is mainly secreted by the kidneys, only small amounts are excreted via faeces. CMC is a cellulose derivative that is already been used as barrier material for adhesion protection with good results regarding efficacy and biocompatibility [10,22-25].

A-Part^® ^Gel does not promote bacterial growth. A-Part^® ^Gel is indicated for use in patients undergoing abdominal or pelvic surgery. A-Part^® ^Gel is intended to reduce the incidence, extent and severity of post-operative adhesions between the abdominal wall and the underlying viscera such as omentum, small bowel, bladder and stomach and between the uterus and surrounding tissues such as tubes and ovaries, large bowel and bladder. A-Part^® ^Gel serves as a temporary absorbable barrier separating the parietal and visceral peritoneal tissue surfaces immediately after surgery. A-Part^® ^Gel acts as a physical barrier for adhesiogenic tissue while the normal tissue repair takes place [26]. After placement on the tissue A-Part^® ^Gel immediately attaches to the surface; A-Part^® ^Gel is elastic, soft and translucent [26]. A-Part^® ^Gel is absorbed and undergoes renal excretion within approx. 6 weeks [18].

### Statistical consideration and sample size estimation

The aim of this study is a first assessment of the safety of the application of A-Part^® ^Gel as an adhesion prophylaxis after major abdominal surgery as compared to control. Sample size considerations are not based on statistical calculations, but on the feasibility of recruitment. It is planned to randomise 60 patients between A-Part^® ^Gel and control, because this is expected to be feasible within six months from a single centre. This phase I study is conducted as a first application of A-Part^® ^Gel in man, and its aim is to receive first safety data as compared to control. The power considerations are based on the specific observation of two major complications of abdominal surgery "occurrence of wound healing impairment or postoperative peritonitis within 28 (+10) days after surgery" as a primary combined endpoint. It is assumed that without application of the A-Part^® ^Gel wound healing impairment will occur with a probability of about 5-10% and postoperative peritonitis will occur with a probability of about 2-5% [31-33]. This leads to the assumption that the combined endpoint will occur in the control group with a probability of about 10-15%. It is the aim of the study to show that the application of A-Part^® ^Gel does not lead to an unacceptable increase in the probability of occurrence of the combined endpoint i.e the non-inferiority of A-Part Gel^® ^as compared to the control. Under the assumption that the application of A-Part^® ^Gel does not increase the probability of the combined endpoint, this study can show at one-sided significance level α = 5% with a power of 80% that the absolute difference of the event probabilities between both treatment groups is not larger than 20-25% [34].

The two-sided 90%-confidence interval for the absolute difference (A-Part^® ^Gel minus control) of probability of occurrence of the primary endpoint will be calculated. If the upper bound of this confidence interval is below 0.25, the hypothesis that the difference is 0.25 or larger will be rejected. Adverse event incidences, wound healing impairment, postoperative peritonitis, adhesion and complication rates will be calculated with two-sided 90% confidence intervals.

The safety analyses will be conducted in the safety population, including all randomized patients with group assignment by treatment received. All analyses will be done with the Statistical Analysis System (SAS).

No interim analysis is planned for this trial.

### Clinical site and safety aspects

Clinical sites are selected according to their experience in abdominal surgery, their clinical research experience, to the number of eligible patients operated in the indicated intervention per year and to their willingness to adhere to clinical trial protocol. The participating centre is mentioned at the end of this paper.

The term "adverse event" covers any sign, symptom, syndrome, illness that appear or worsen in a patient during the period of observation in the clinical trial and that may impair the well-being of the subject. The term also covers laboratory findings of other diagnostic procedures that are considered to be clinically relevant.

A "serious adverse event" is any event occurring at any time during the period of observation that results in death, is immediately life - threatening, requires or prolongs hospitalization, results in persistent or significant disability and incapacity.

All SAEs are reported on a SAE report form and must be sent by fax within 24 hours to the sponsor. The sponsor will notify the competent authorities, ethics committees and all other potential investigators about the SAEs in line with applicable regulatory requirements.

Analysis of safety related data performed with respect to:

- Frequency of SAEs

- Frequency of SAEs stratified by severity

- Frequency of SAEs stratified by causality.

### Trial organization, quality control, registration and ethical aspects

The trial is initiated and sponsored by B|Braun Aesculap and conducted by Aesculap AG in cooperation with the Clinical Trials Centre (ZKS) in Freiburg, Germany. The Clinical Trials Centre (ZKS) in Freiburg is responsible for biometry, data base and project management. Monitoring is done by an authorized, qualified, representative person of Conventis AG who will visit the investigational site at regular intervals to verify adherence to the protocol and legal requirements, perform source data verification and assist the investigator in his related activities. The sponsors role is limited to supplying the participating centre with the medical device and surgical support. The sponsor is not involved in the data base management. He has taken out an insurance policy to cover all patients taken part in the trial.

The trial is coordinated by the Clinical Trials Centre (ZKS) in cooperation with B|Braun Aesculap. Aesculap AG has registered the trial on 13^th ^November 2008 at the clinical trials register (Identifier Number NCT00646412, http://www.clinicaltrial.gov). Before the start of the trial the independent ethics committee of the Ludwig-Maximilians-University (LMU) of Munich gave their approval on 3^rd ^April 2008. The trial is performed according to the Declaration of Helsinki in its current German version, the national medical device law and the guidelines for Good Clinical Practice (GCP).

### Data management and quality assurance

Data documentation will be performed on study-specific case report form (CRF) consisting of 3-layer NCR paper, which form a front end to the database. SAS software will be used to review the data for completeness, consistency and plausibility.

The clinical data base includes all information until 3 months post-operatively and will be closed after the 3 months follow-up visit of the last patient randomised into the trial.

### Current status and duration of the trial

The study protocol for the trial was completed on 13^th ^March 2008. After completion of the contract the study was initiated on 9^th ^July 2008. One centre recruited patients with the goal of 60 patients. After receiving the positive ethics approval the first patient was randomized on 28^th ^July 2008. The recruitment was completed in December 2009.

## Discussion

Adhesions between visceral organs and the abdominal wall are frequent sequelae of abdominal or pelvic surgery, causing complications such as small bowel obstruction or infertility [2,8,23]. Safe, efficient and easily to apply barrier measures are still missing. Hyaluronic acid carboxymethylcellulose films, oxidized regenerated cellulose and extended polytetrafluoroethylene film have been used to prevent adhesions after surgical trauma [2]. The use of a PVA membrane gave quite promising results clearly better than other commercially available products [26]. Membranes are not applicable in every surgical site. Therefore a gel or a liquid solution is preferred in the prevention of post surgical adhesions.

Anti-adhesion barriers basically fall under two main categories: macromolecular solutions (crystalloids, polysaccharide, hyaluronic acid, fibrin, polyethylene glycol, phospholipids) and mechanical devices (oxidized-regenerated cellulose, hyaluronic acid-carboxycellulose, expanded polytetrafluoroethylene and autologous transplants).

Crystalloid solutions are proposed to work by hydro-floatation to separate raw peritoneal surfaces. But they have a negative effect on post-operative adhesions [35], because the volume of the applied crystalloids are absorbed very rapidly [36]. By hydro-floatation and siliconization of intra-abdominal structures with polysaccharide such as Dextran solutions a physiological separation occurs between peritoneal surfaces. Data of Dextran solution used as a barrier are discussed controversial. There are studies showing good anti-adhesive results but other studies failed to demonstrate any clinical improvement and instead showed severe side effects such as edema, ascites and coagulopathy [37,38].

Carboxymethylcellulose (CMC) is a derviate of cellulose and works by separating raw surfaces and allowing independent healing of traumatized peritoneal surfaces [39,40]. CMC showed a good anti-adhesive effect in experimental models but not in clinical trials. CMC in membranes together with hyaluronic acid and CMC membranes with polyethylene oxide and calcium chloride have also been reported as effective anti-adhesive agents in clinical trials [41].

Oxidized regenerated cellulose (ORC) is another barrier used for the prevention of adhesions [42]. When applied to raw peritoneal surface, it gels within 8 h [43]. Experimental and clinical models reported good preventive results by forming a barrier which physically separates the adjacent raw peritoneal surfaces. However poor results were noted in the presence of blood and probably also at coexisting infections [44]. ORC is not widely used in general surgery today.

The experimental effects of a glucose polymer have been promising, reducing adhesions without obvious side effects [45,46]. In a RCT it was shown that this polymer reduces the incidence of adhesions and that its application is safe and easy [46]. However, a Meta-analysis report on pelvic adhesion, has not recommended this polymer for the prevention of intra-abdominal adhesions [47].

Hyaluronic acid (HA) is a naturally occurring glycosamin which is biocompatible, non immunogenic and bioabsorbable, therefore it seems to be well suited as an anti-adhesive agent [48]. Compounds consisting of HA act as a barrier because they coat serosal surfaces and provide a certain degree of protection from serosal desiccation and other types of injury. But they have also been shown to have an anti-inflammatory effect [49-51]. The most popular product on the market is Seprafilm^®^, a combination of HA and CMC. Seprafilm^® ^reduces adhesive formation, but it is very expensive [13]. Its use increases the risk of anastomotic deshiscence, the formation of abscess [23] and inflammatory reaction [52].

In a previous randomized controlled trial the safety and efficacy of another anti- adhesive agent, Intergel^® ^was evaluated in colorectal surgery [53]. Intergel^® ^is a hyaluronic based gel which is cross-linked with carboxylate groups by chelation with ferric (Fe^3+^) ions. The aim of the study was to reduce the occurrence of intestinal obstructions due to abdominal surgery using Intergel^®^. Patients who received Intergel^® ^showed a high incidence of anastomotic dehiscence and a prolonged postoperative ileus in comparison to the control group treated only with distilled water. Furthermore, wound infections were observed more often in the Intergel^® ^group. Due to this high incidence of adverse effects in the Intergel^® ^treated group the study was prematurely terminated. The authors discouraged from the use of Intergel^®^, especially during intervention in which the gastrointestinal tract has been opened, because the application of Intergel^® ^may interfered with the anastomotic wound healing process.

If surfaces are damaged, another way to prevent adhesion formation is the application of phospholipids. Experimental results have been effective using phospholipids, mainly phospatidylcholine, both in models with peritonitis and under normal conditions. No negative effects were seen on wound healing or anastomosis safety [45,54]. However no clinical studies have been performed so far.

Polyethylene glycol has also been used as a barrier to prevent post surgical adhesions. Experimental studies showed good results and clinical trials reported an adhesion-preventing effect in gynaecological surgery. Additionally, no effect has been noted on pregnancy rates after treatment [54]. In contrast, a Meta-analysis failed to demonstrate the evidence for the use of polyethylene glycol in the prevention of post surgical adhesions [47].

Fibrin sealant are compounds of fibrinogen and thrombin. They have the advantage of decreasing bleeding and increasing the production of plasminogen activator (PA) and plasminogen activator inhibitor-1 (PAI) which may be beneficial in the prevention of adhesions [55]. But experimental results are diverging and no clinical data are available [56,57].

Non-dissoluble membranes of expanded polytetrafluoroethylen (ePTFE) have been used successfully to reduce adhesions. But these membranes are difficult to apply and must be fixed in place usually with sutures. In addition they are non biodegradable and should be removed at a later time raising the possibility of damage and subsequent adhesion formation during this surgical episode [2]. One clinical study has been conducted, showing a reduction in postoperative adhesions [58]. But ePTFE is not widely used as a barrier in surgery at present.

No clinical data are available for the use of collagen films as a barrier but experiments reported an effect against adhesion formations [59]. Transplantation of an autologous mesothelial cell sheet has recently shown positive experimental results and offers also a new interesting platform for future development.

None of these agents mentioned above have been proven to be uniformly efficacious and safe under all surgical conditions. Despite initial promising results in postoperative adhesion prevention, none of them have become a standard application. Therefore there is still the need of a barrier that is safe and efficient in reducing the incidence and the extent of surgical adhesion occurring after general abdominal surgical procedures.

A-Part^® ^Gel consists of PVA and CMC and shows no cytotoxity, no allergenic reactions, no systemic toxicity and no genotoxicity [13]. Physical properties such as viscosity and adherence to the wound have also been tested with good results [26].

In this study A-Part^® ^Gel is used as a physical barrier between the injured peritoneal surface to prevent post surgical adhesions. Animal studies have shown high efficiency in the prevention of adhesions [26,27,60,61]. Wound healing and anastomosis healing were not negatively affected by the application of A-Part^® ^Gel in these animals.

To explore the efficacy of A-Part Gel^® ^in reducing adhesion in humans, adhesion to the abdominal wall will be assessed by ultrasound examination. The gold standard of evaluating adhesion formation by second look assessment is only possible in surgical procedures that require a planned re-operation. However, as this limitation would reduce the amount of eligible patients this assessment is not being chosen, especially as there would be no benefit for the primary objective of this trial as a safety study. Assessment of adhesion development will be done by ultrasound examination. Sigel *et al. *as well as Steitz *et al. *[62,63] described a technique for non-invasive ultrasound examination to detect and map abdominal adhesions. The findings that identify abdominal wall adhesions are based on the presence or restriction of ultrasonically observed movement of abdominal viscera in reference to abdominal wall. Sliding is the result of forces applied by respiratory excursions or by manual ballottement of the abdominal wall and is referred to as viscera slide. In brief, examination of spontaneous visceral slide with ultrasound scanning, provide information on the presence or absence of adhesions. Normal and abnormal visceral slide can be distinctly defined by simple measurement of excursion distance. Restricted visceral slide has been defined as reduced movement at a given point on the abdominal wall during ultrasound scanning of spontaneous visceral slide produced by regular and deep respirations or during longitudinal and transverse scanning of induced viscera slide produced by manual compression. It is strongly correlated with the presence of abdominal adhesions as several authors confirm. Most studies report a sensitivity of 90% and a specificity between 86-93% [62-64]. The method has only minor shortcomings in the lower third of the abdomen and may not detected a long thin adhesion or adhesions to the movable omentum, probably because this type of adhesion would not cause a significant restriction of viscera movement [65]. For assessment at the laparotomy scar spontaneous visceral slide is the most sensitive indicator of adhesions [62,63,66].

## Conclusions

This prospective, randomised, controlled, patient - and observer- blinded, single centre study evaluates as a primary endpoint, if the application of A-Part^® ^Gel is safe as adhesion prophylaxis, in comparison to a non-treated control group, during and after abdominal surgery.

## Abbreviations

AE: Adverse Event; SAE: Serious Adverse Event; PVA: Poly Vinyl Alcohol; CMC: CarboxyMethylCellulose; CRF: Case Report Form; GCP: Good Clinical Practice; MOF: multiple organ failure; ZKS: Clinical Trials Centre; RCT: Randomized Controlled Trial; HA: Hyaluronic acid; ePTFE: expanded polytetrafluoroethylene; ORC: Oxidized regenerated cellulose; PA: Plasminogen activator; PAI: Plasminogen activator inhibitior; INSECT: interrupted or continuous slowly absorbable sutures - evaluation of abdominal closure techniques.

## Competing interests

This study and its publication is sponsored by Aesculap AG, Tuttlingen, Germany.

## Authors' contributions

PB and HPK (B|Braun Aesculap, Tuttlingen, Germany) managed and conducted the trial together with the Clinical Trials Centre, Freiburg and KWJ. The study protocol was written by CW and EO together with the Clinical Trials Center, Freiburg. PB and HPK wrote together with RL the manuscript. CS is involved as an biostatistician. All authors have read and approved this manuscript.

## Pre-publication history

The pre-publication history for this paper can be accessed here:

http://www.biomedcentral.com/1471-2482/10/20/prepub
